# Palliative transpedicular partial corpectomy without anterior vertebral reconstruction in lower thoracic and thoracolumbar junction spinal metastases

**DOI:** 10.1186/s13018-015-0255-z

**Published:** 2015-07-17

**Authors:** Chien-Chun Chang, Yen-Jen Chen, Da-Fu Lo, Hsien-Te Chen, Horng-Chaung Hsu, Ruey-Mo Lin

**Affiliations:** Department of Orthopedic Surgery, China Medical University Hospital, No. 2, Yuh-Der Road, Taichung, 404 Taiwan; Department of Orthopedic Surgery, School of Medicine, China Medical University, Taichung, Taiwan; Department of Orthopedic Surgery, Tainan Municipal An-Nan Hospital, Tainan, Taiwan

**Keywords:** Anterior vertebral reconstruction, Cancer, Implant failure, Partial corpectomy, Posterior instrumentation, Posterolateral transpedicular approach, Spinal metastasis, Stability, Survival rate, Thoracolumbar junction tumor

## Abstract

**Background:**

The thoracolumbar junction is the transition from a stiff (thoracic spine) to a mobile zone (lumbar spine) and is relatively unstable compared with the thoracic and lumbar portions of the spine. The need for anterior reconstruction after a corpectomy has been emphasized by several authors. However, for patients with a relatively short life expectancy, anterior reconstruction may be unnecessary. Posterior instrumentation alone may be sufficient to provide pain relief and stability for such patients. The goal of this study was to assess the postoperative outcomes and survival rates of patients with tumor metastases of the lower thoracic spine and thoracolumbar junction (T10–L1) who underwent transpedicular partial corpectomy without anterior vertebral reconstruction.

**Methods:**

From November 2001 to February 2015, 29 patients diagnosed with symptomatic spinal cord compression caused by tumor metastasis involving T10 to L1 underwent palliative surgery that involved a posterolateral transpedicular partial corpectomy without anterior reconstruction. The surgical indication was neurologic progression. A follow-up was conducted for all of the patients, including reviewing medical records and performing an examination in the outpatient department.

**Results:**

The patients ranged in age from 33 to 83 years (mean, 61.6 years). Neurologic improvement by at least one Frankel grade was noted in 75.9 % of the patients (*N* = 22). Neither intraoperative mortality nor implant failure was reported. The median survival rate was 7.43 months (range, 0.47–28 months).

**Conclusion:**

The results of this study suggest that the stability of implants can be maintained up to 28 months with satisfying functional outcome after a palliative posterolateral transpedicular partial corpectomy without anterior reconstruction.

## Background

Surgery for treating spinal metastatic disease offers more benefits in walking ability and reduces the need for opioid analgesia compared with radiation therapy alone [1]. The traditional approach for treating spinal metastasis is to use posterior procedures for decompression followed by anterior column reconstruction. The need for anterior column reconstruction after tumor excision has been emphasized by many previous studies [2–4]. However, the need for anterior reconstruction has been challenged by other authors who have treated thoracic spine metastatic disease and have hypothesized that the thoracic spine has more stability than other spinal segments, making anterior reconstruction unnecessary [5]. This finding raises the question if anterior reconstruction is also unnecessary in other spine segments during treatment of carefully selected patients.

As we know, the thoracolumbar junction (TLJ) of the spine is considered the most mobile spinal segment [6], and the need for anterior reconstruction after tumor excision is theoretically more important than for other spinal segments. However, in patients with a short life expectancy, anterior reconstruction may be unnecessary and may be associated with severe morbidity and is also contraindicated in patients whose general condition is poor. Posterior instrumentation alone may provide sufficient stability and the advantage of improved pain relief.

The goals of this study were to evaluate the clinical outcomes of patients who underwent transpedicular partial corpectomy without anterior reconstruction in spinal metastases of the lower thoracic spine and TLJ (T10–L1).

## Methods

This was a retrospective review of our spine-tumor database from November 2001 to February 2015 that identified 29 consecutive patients who underwent surgery for metastatic spinal diseases involving T10 to L1 by a single surgeon in our hospital. The research ethics committee of our hospital approved this retrospective analysis. All of the patients underwent a posterolateral transpedicular partial corpectomy without anterior vertebral reconstruction.

The clinical data were collected by reviewing medical records, including patient age, sex, date of operation, date of death or final follow-up, tumor histology, and preoperative and postoperative ambulatory status graded using the Frankel system. The overall survival rate was calculated from the date of surgery to the date of final follow-up or death. The survival curve was calculated using the Kaplan-Meier method.

The clinical indication for operation was symptomatic metastatic spinal cord compression with neurologic progression. A multidisciplinary team evaluated all the patients to determine their suitability to undergo surgery. Preoperative evaluation included laboratory data, plain radiography, and magnetic resonance imaging.

Posterior spinal instrumentation was performed for all the cases after adequate decompression. Local radiotherapy, targeted therapy, and systemic chemotherapy were performed according to appropriate cancer guidelines after the operation. Wound healed typically 3 to 4 weeks after the operation.

### Technique

#### Approach

The patients were positioned prone on a Jackson spine table. A midline incision was made at least two levels above and below the tumor to expose the tumor adequately.

#### Decompression

Total laminectomy was performed one level above and below the affected segment. Facetectomies and pedicle resection for the affected vertebra were performed. We removed the posterior and lateral epidural tumor after ligation of the nerve roots at the affected vertebra (thoracic spine only). The tumor in the posterior vertebral body was removed after identification of the plane between the posterior longitudinal ligament and the dura in order to achieve adequate circumferential decompression. A partial corpectomy was performed and generally involved removing less than 50 % of the vertebral body. The goals of the surgery were adequate decompression and palliative tumor excision, not en bloc resection.

#### Instrumented stabilization

Instrumentation with pedicle screws was performed two levels above and below the lesion. Anterior vertebral body reconstruction and posterolateral fusion with bone graft were not performed in any of the patients.

## Results

The patients ranged in age from 33 to 83 years old (mean, 61.6 ± 11.6 years, 16 men, 13 women). The mean operating time was 4.6 ± 0.93 h. The mean intraoperative blood loss was 1181 ± 657.6 ml. The most common metastatic tumor in our cases was lung cancer, as shown in Table 1.

**Table 1:** Demographic data of the 29 patients who underwent transpedicular partial corpectomy without anterior vertebral reconstruction for metastatic thoracolumbar diseases

					Frankel grade	
Case no.	Age (years), sex	Primary tumor	^a^Location	Survival days	Preoperative	Postoperative
1	75, M	Lung	T11, L1	223	C	C
2	69, M	Lung	T12	23	C	C
3	59, F	Lung	T11, 12	626	D	E
4	73, F	Lung	T11, 12	265	C	E
5	81, M	Lung	L1	115	C	D
6	51, M	Lung	T10	171	C	C
7	33, F	Neuroendocrine	T12	98	C	D
8	65, F	Lung	L1	422	C	E
9	69, M	Lung	T10	61	C	D
10	51, F	Lung	T12, L1	803	C	E
11	76, M	Lung	L1	73	C	C
12	73, F	Cervical	T12, L1	256	D	E
13	51, M	Esophagus	L1	14	C	C
14	68, M	Liver	L1	110	B	C
15	44, F	Adrenal gland	T11	325	B	D
16	83, F	Liver	T12	45	C	C
17	67, M	Kidney	L1	43	C	C
18	55, F	Kidney	T10	169	B	C
19	55, M	Lung	T11	633	C	E
20	64, F	Lung	T12, L1	299	D	E
21	65, F	Multiple myeloma	T10	450	C	D
22	43, F	Cervical	T11	73	D	E
23	49, M	Pancreas	L1	^b^851	C	E
24	59, M	Lung	T12, L1	^b^829	C	E
25	55, M	Lung	T11	104	C	C
26	49, M	Rectus	T11, 12	185	C	E
27	58, M	Lung	T11	103	C	D
28	70, M	Prostate	T12	^b^484	D	E
29	62, F	Multiple myeloma	T11	^b^432	C	D

^a^“Location” means the segment where we performed the operation

^b^Cases of numbers 23, 24, 28, and 29 were still alive at the time of most recent contact

Neurologic improvement was noted in 22 of 29 patients (75.9 %). Overall, 65.5 % of the patients (19 of 29) could walk after the operation. After the operation, no patient showed deterioration of the functional scales measured by Frankel grading (Table 2). Fourteen of 24 (58.3 %) nonambulatory (Frankel level B/C) patients became ambulatory (Frankel level D/E) again. For patients who survived more than 6 months, 93.3 % (14 of 15) were ambulatory.

**Table 2:** Functional status: preoperative versus postoperative Frankel grading

Preoperative grading	Postoperative grading				
	A	B	C	D	E
A					
B			3	1	
C			7	6	7
D					5
E					

Figures within boxes indicate the numbers of patients with each functional status

**Fig. 1:**
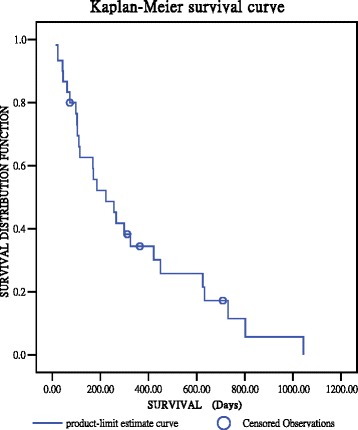
Kaplan-Meier survival curve of the 29 patients who underwent transpedicular partial corpectomy without anterior reconstruction for metastatic thoracolumbar diseases

Postoperative complications are listed in Table 3. Neither intraoperative mortality nor cases requiring revision surgery were reported. Among the seven complications, only one was related to surgery. Two patients died within 30 days of the operation. One patient died because of nonsurgery-related pneumonia and sepsis. The other patient died because of respiratory failure caused by lung cancer. The median postoperative survival time was 7.43 months (range, 0.47–28 months). The Kaplan-Meier curve (Fig. 1) shows that the cumulative survival rate was 52.2 % at 6 months and 30.1 % at 1 year. Among the patients, nine survived for more than 1 year. Three patients survived more than 2 years. Four patients were still alive at the time of this report, with follow-up times ranging from 14 to 28 months.

**Table 3:** Postoperative complication

Complication	No. of patients
Neurologic progression	0
CSF leakage	1
Wound dehiscence/infection	0
Intraoperative mortality	0
Respiratory failure	1
GI bleeding/gastrointestinal perforation	2
Pulmonary embolism	0
Revision surgery	0
Local recurrence	1
30-day mortality	2

**Fig. 2:**
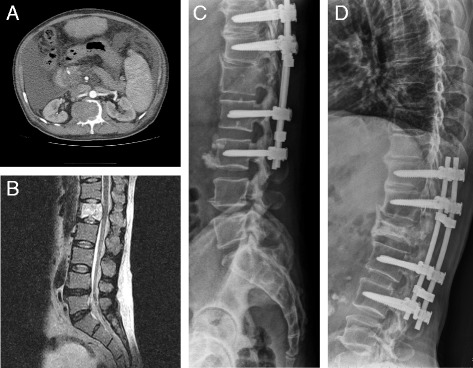
Preoperative and postoperative images of a 49-year-old man with L1 vertebral metastatic spinal cord compression from pancreatic cancer who underwent surgery using the posterolateral transpedicular approach without anterior vertebral reconstruction. The CT angiography (**a**) and MRI image (**b**) demonstrate metastatic spinal tumor with cord compression at L1. The immediate postoperative radiographs (**c**) and the 28-month follow-up radiographs (**d**) demonstrate no screw loosening or implant failure

No implant failure was noted within the follow-up time (mean follow-up, 9.52 ± 8.37 months). The implants were maintained without loosening or failure up to 28 months without reconstruction (Fig. 2).

## Discussion

The posterolateral transpedicular approach (PTA) with circumferential decompression and reconstruction has become widely accepted in treating spinal metastasis, especially in cases with circumferential tumor compression or multilevel spinal involvement. Adequate decompression and stabilization are the goals of this surgery. The need for anterior reconstruction for adequate stabilization in the PTA has been emphasized by several authors [2–4]. Two biomechanical studies have demonstrated that circumferential spinal fixation provides superior construct stability compared with anterior or posterior fixation alone [7, 8].

Anterior reconstruction is not completely without risk. Akeyson et al. studied 25 patients who underwent a single-stage complete spondylectomy, vertebral body reconstruction, and posterior segmental spinal stabilization for malignant metastatic disease of the thoracolumbar spine [9]. The overall complication rate was 48 %, and the most severe complications were related to migration of the material used for reconstruction. Anterior reconstruction during PTA with polymethylmethacrylate replacement may have a 16 % dislodgement rate and may require revision surgery [9]. Reconstruction with the Steinmann pin and cement also showed a complication rate of approximately 14.3 % [10]. Even when a modern expandable cage was used, complications were as high as 10–21 % [11–14]. Anterior vertebral reconstruction during PTA also increases blood loss and operation time and may increase morbidity and complications. These factors raise considerable concerns for patients with metastatic tumors and poor general condition because they may result in delayed treatment with chemotherapy/target therapy or radiotherapy.

T10–L1 is a transition from the stiff (thoracic spine) to the mobile zone (lumbar spine) and is relatively unstable compared with the thoracic and lumbar spine. The TLJ is a frequent site of spinal trauma, and up to 67 % of operative cases involve TLJ traumatic injuries [6]. Previous biomechanical studies have demonstrated that anterior column integrity determines the risk of sagittal collapse and kyphosis at the thoracolumbar spine [15]. Oda et al. studied the stability of five reconstruction methods after total spondylectomy in cadaveric spines and concluded that short circumferential instrumentation provided more stability than did multilevel posterior instrumentation alone [16]. According to these studies, anterior column reconstruction is crucial in treating lower thoracic and TLJ spinal fractures, and, basically, treating metastatic spinal tumors involving T10–L1 should also follow this rule. However, the complications of anterior reconstruction are an important concern in treating spinal metastases.

Previous studies have reported the results of the PTA without anterior reconstruction, but no study had directly focused on tumors involving T10–L1. Bridwell et al. [17] studied 25 patients with metastatic spine disease treated with PTA and posterior segmental spinal instrumentation and reported no cases of implant failure; however, only seven cases involved T10 to L1. Walter et al. studied 57 consecutive patients with metastatic vertebral tumors who were treated using PTA without anterior reconstruction [18]. The complication rate was only 5.3 %, including one seroma and two superficial wound infections. No implant failure was reported. However, the study by Walter et al. involved only ten cases at T10–L1. Cho et al. [19] studied 21 consecutive patients with metastatic tumors who were treated using PTA without anterior reconstruction and reported a complication rate of 19 % (4 of 21). However, Cho’s study involved only 13 cases at T10–L1. Based on our research, no previous studies have directly focused on metastatic tumors involving T10 to L1 treated using PTA without anterior reconstruction.

In this study, no cases of implant loosening or failure were noted by follow-up radiography (mean follow-up, 9.52 ± 8.37 months), and three patients survived more than 2 years without implant failure or loosening.

The greatest concerns about using PTA without anterior reconstruction are the durability and stability of posterior instrumentation. We attempted to resolve this problem using two methods. First, no more than 50 % of the vertebral body was resected. Bilsky et al. [20] suggested that vertebral body reconstruction of the spine may be unnecessary if less than 50 % of the vertebral body has been resected. These methods can achieve adequate decompression without sacrificing stability. Second, all of the patients in this study underwent instrumentation two levels above and below the tumor lesion after the PTA. Some biomechanical studies have demonstrated that multilevel fixation (long constructs) have proved to be more reliable and effective in treating thoracolumbar injuries—with or without anterior reconstruction—compared with short-segment fixation [14, 15]. If the patient’s life expectancy is longer than 2 years, or when stability is a concern, further anterior reconstruction may be attempted later.

The goals of the palliative operation in treating metastatic spine tumor are to decompress the tumor for functional improvement and to stabilize the spine structure for pain relief rather than en bloc resection of the tumor. With fewer postoperative complications, patients can accept further oncologic therapies (including radiotherapy, chemotherapy, and targeted therapy) more quickly and may have better functional status and quality of life. Because of the complications associated with anterior reconstruction, posterior instrumentation alone seems to be more suitable for selected patients. These patients can recover more quickly and smoothly when this less invasive method is used. Earlier systemic oncologic therapies and radiotherapy may lower the recurrence rate of the metastatic spinal tumor. With this method, only one local recurrence of previous metastatic spinal tumor was noted in our series.

There are still some limitations of this study. First, this was a retrospective study. Second, the sample size is relatively small because we focused on the level of T10 to L1. Third, this study did not have a control group. Although the article lacks a control group to compare against, we believe that the rate of neurological improvement using these methods is not inferior to addition of anterior column reconstruction. The neurological improvement rate of corpectomy with anterior reconstruction ranges from 29.6 to 61.5 % [10, 12, 13]. In our study, neurological improvement was noted in 22 of 29 patients (75.9 %). According to these studies, we believe that PTA without anterior reconstruction provides a comparable rate of neurological improvement and less complication compared with circumferential decompression and anterior reconstruction. With the principles mentioned above, patients with symptomatic tumor metastasis involving T10 to L1 can be treated simply with palliative PTA and posterior instrumentation without anterior reconstruction with satisfying functional results.

## Conclusions

The PTA without anterior reconstruction is an option for treating T10–L1 metastases. The results of this study revealed that applying these principles and methods can maintain implant stability for up to 28 months without anterior vertebral reconstruction in patients with T10–L1 metastases.

## Abbreviations

PTA: posterolateral transpedicular approach
TLJ: thoracolumbar junction
